# Green Biosynthesis, Antioxidant, Antibacterial, and Anticancer Activities of Silver Nanoparticles of *Luffa acutangula* Leaf Extract

**DOI:** 10.1155/2021/5125681

**Published:** 2021-09-29

**Authors:** Devi Nallappan, Agustine Nengsih Fauzi, Balam Satheesh Krishna, Basivi Praveen Kumar, Avula Vijaya Kumar Reddy, Tasqeeruddin Syed, Cirandur Suresh Reddy, Nik Soriani Yaacob, Pasupuleti Visweswara Rao

**Affiliations:** ^1^Bioindustrial Technology Program, Faculty of Agro-Based Industry, Universiti Malaysia Kelantan, Campus Jeli, 17600 Jeli, Malaysia; ^2^Department of Biomedical Science, Faculty of Medicine, University of Malaya, 50603 Kuala Lumpur, Malaysia; ^3^Department of Chemical Pathology, School of Medical Sciences, Universiti Sains Malaysia, 16150 Kubang Kerian, Kelantan, Malaysia; ^4^Department of Chemistry, Sri Venkateswara University, -517502, Tirupati, Andhra Pradesh, India; ^5^Department of Biochemistry, Sri Kadiri Babu Rao College of Agriculture, C.S. Puram, Andhra Pradesh-523112, India; ^6^Centre for Chemical Sciences and Technology, Institute of Science & Technology, Jawaharlal Nehru Technological University Hyderabad, -500085, Hyderabad, Telangana, India; ^7^Department of Pharmaceutical Chemistry, College of Pharmacy, King Khalid University, Abha-61421, Saudi Arabia; ^8^Department of Biomedical Science and Therapeutics, Faculty of Medicine and Health Sciences, Universiti Malaysia Sabah, Kota Kinabalu 88400, Malaysia; ^9^Department of Biochemistry, Faculty of Medicine and Health Sciences, Abdurrab University, Jl Riau Ujung No. 73, Pekanbaru, 28292 Riau, Indonesia; ^10^Centre for Excellence in Biomaterials Engineering (CoEBE), AIMST University, 08100 Bedong, Kedah, Malaysia

## Abstract

Studies on green biosynthesis of newly engineered nanoparticles for their prominent medicinal applications are being the torch-bearing concerns of the state-of-the-art research strategies. In this concern, we have engineered the biosynthesized *Luffa acutangula* silver nanoparticles of flavonoid *O*-glycosides in the anisotropic form isolated from aqueous leave extracts of *Luffa acutangula*, a popular traditional and ayurvedic plant in south-east Asian countries. These were structurally confirmed by Ultraviolet-visible (UV-Vis), Fourier transform infrared spectroscopy accessed with attenuated total reflection (FTIR-ATR) spectral analyses followed by the scanning electron microscopic (SEM) and the X-ray diffraction (XRD) crystallographic studies and found them with the face-centered cubic (*fcc*) structure. Medicinally, we have explored their significant antioxidant (DPPH and ABTS assays), antibacterial (disc diffusion assay on *E. coli*, *S. aureus*, *B. subtilis*, *S. fecilis*, and *S. boydii*), and anticancer (MTT assay on MCF-7, MDA-MB-231, U87, and DBTRG cell lines) potentialities which augmented the present investigation. The molecular docking analysis of title compounds against 3NM8 (DPPH) and 1DNU (ABTS) proteins for antioxidant activity; 5FGK (Gram-Positive Bacteria) and 1AB4 (Gram-Negative Bacteria) proteins for antibacterial activity; and 4GBD (MCF-7), 5FI2 (MDA-MB-231), 1D5R (U87), and 5TIJ (DBTRG) proteins for anticancer activity has affirmed the promising ligand-protein binding interactions among the hydroxy groups of the title compounds and aspartic acid of the concerned enzymatic proteins. The binding energy varying from -9.1645 to -7.7955 for Cosmosioside (1, Apigenin-7-glucoside) and from -9.2690 to -7.8306 for Cynaroside (2, Luteolin-7-glucoside) implies the isolated compounds as potential bioactive compounds. In addition, the performed studies like QSAR, ADMET, bioactivity properties, drug scores, and toxicity risks confirmed them as potential drug candidates and aspartic acid receptor antagonists. This research auxiliary augmented the existing array of phytological nanomedicines with new drug candidates that are credible with multiple bioactivities.

## 1. Introduction

Phytological origin is the main source for many flavonoids and corresponding flavonoid *O*-glycosides (FOGs) [1]; these FOGs are proven antioxidant [2], antimicrobial [3], anticancer [4], antiobesity [5], and medicinal agents [6]. The collective advances of FOGs concerning nanotechnology have emerged as a new arena that captivating medicinal researchers to pursue research in it [7, 8]. As acclaimed *in vivo* oxidation being identified as a vital process that spawns the ample energy for the proper execution of biological processes in all organisms, often, it causes the overproduction of free radicals in turn for the cell damage and in turn for the metabolic diseases like diabetes, cardiovascular diseases, cancers, and neurological disorders [9, 10]. In its counter administration, antioxidants inhibit the reactive free radicals by neutralizing and arrest the structural disruption of biomolecules in cells [11].

During so, the distinguished biosynthesized phytological nanoparticles (NPs) are identified more remarkable than their plant extracts in exhibiting potential activity [12]. This has fascinated nanotechnology in embodiment with scientific results that abridged the gap between with atomic/molecular structures and bulk materials and accelerated the chemotherapeutic potency in treating various diseases [13, 14]. Structurally, the surface-to-volume ratio of NPs is contrarily proportional to their sizes, [15] more precisely the inherent potential of silver nanoparticles (AgNPs) grows reciprocally with an escalation in the specific surface area owing to high surface energy and catalytic reactivity ^16^. The preference and advancement of green chemistry over conventional are due to eco-friendliness, cost-effectiveness, and feasibility for large-scale synthesis [16, 17]. The extensive array of AgNPs with medicinal efficacies like anticancer, [18, 19], antioxidants [20], and antimicrobial [21] abilities is derived from various plant origins like *Rhinacanthus nasutus* [22], *Trigonella foenum-graecum* [23], *Ocimum bacillicum* [24], *Vitex negundo L* [25], *Hypnea musciformis (Wulfen) JV lamouroux* [26], *Terminalia chebula* [27], *Raphanus sativus* var. *aegyptiacus* [28], *Citrus sinensis* [29], *Cassia roxburghii* [30], *Eurocyma longifolia* [31], *Annona muricata* [32], and *Eriobotrya japonica* [33].

It is profound that excessive usage of antibiotics results in dissemination and emergence of multidrug-resistant strains of several types of microorganisms [31, 34]. In this scenario, the needs and demands to discover new medicinal agents are increasing, and nanotechnology paves ways to synthesize NPs to substitute current antibiotics and other synthetic agents. In such, *Luffa acutangula* (*LA*), a traditional perennial flowering climber plant, ordinarily stated as ridge gourd regards to *Cucurbitaceae* family and is enriched with medicinal properties [35] like antioxidant, antidiabetic, antiproliferative, antiangiogenic, anticataleptic, analgesic, antiulcer, and antimicrobial activities [36, 37].

**Figure 1:**
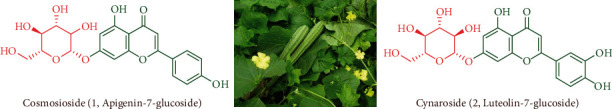
Potential natural FOGs (1 and 2) identified in *Luffa acutangula* (LA) leaves.

As LA plant parts are enriched with a large number of pharmacologically active phytochemicals like flavonoids, proteins, saponin triterpene, anthraquinones, fatty acids, and other phytoconstituents, it is ethnopharmacologically used to treat hemorrhoids, leprosy, splenitis, and ringworm infections by topical administration of pulverized leaves of LA [38]. Hence, we synthesised LAAgNPs from the leaf extract of LA and succeeded in synthesising AgNPs. The flavonoids present in leaves *viz.*, Cosmosioside (1, Apigenin-7-glucoside), Cynaroside (2, Luteolin-7-glucoside) with potential antioxidant, antibacterial, and anticancer activities are FOGs originated from *O*-glycosidic linkage of (2*ξ*)-*β*-*D*-arabino-Hexopyranose with 7-OH group of Apigenin and *O*-glycosidic linkage of *β*-*D*-glucopyranose with 7-OH group of Luteolin (Figure 1).

## 2. Materials and Methods

### 2.1. Chemistry

Silver nitrate (AgNO_3_) and 2, 2-diphenyl-1-picrylhydrazyl (DPPH) were procured from Sigma (St. Louis, Missouri MO, United States). All the other chemicals were of analytical grade. Human glioma cells (DBTRG and U87) and human breast adenocarcinoma cells (MCF-7 and MDA-MB-231) were procured from the American Type Cell Culture (ATCC). The media, serum, and antibiotics were procured from Gibco™ (Melbourne, Australia).

### 2.2. Collection of Plant Materials

Fresh leaves of LA were collected from the botanical garden, Department of Botany, Universiti Malaysia Kelantan, Campus Jeli, Malaysia. The collected plant material was rinsed under running tap water to remove all attached materials. The plants and its components have been collected according to the guidelines.

### 2.3. Preparation of Plant Extract and Synthesis of LAAgNPs

The leaves were shade dried and powdered. Three different concentrations of leaf extracts were prepared (1.0, 2.5, and 5.0%). The mixture was boiled in water bath continuously for 30 min at 100°C and filtered through Whatman No. 1 filter paper, and the same filtrate was used as reducing agent during the nanoparticle synthesis. A stock solution of 1 mM AgNO_3_ was prepared. LA leaf extracts (25 mL) were added to 25 mL of AgNO_3_ solution in 1 : 1 ratio in the dark conditions. The colour changes and was observed from light colour to dark colour, and the synthesis of nanoparticle was confirmed by using UV-Vis spectroscopy. The solution was centrifuged at 8000 rpm for 10 min. The pellets of formed silver nanoparticles were dried and powdered for further use.

### 2.4. Characterization of LAAgNPs

The solution that contained colloidal silver nanoparticles was diluted 10 fold using into distilled water. The reduction of pure silver ions was monitored in the range of 200-700 nm in the visible region by measuring the UV-Vis spectrum of the colloidal solution obtained at different functional time. The crystallization nature of the silver nanoparticles was analysed by using XRD crystallography. The functional groups associated with silver nanoparticles analysis were done by FTIR-ATR spectroscopy. The FTIR spectrum was measured at the adsorption range of 500-4000 cm^−1^. The particle size was determined by using scanning electron microscope (SEM). The thin layer of synthesised silver nanoparticles was mounted on a copper grid coated with carbon. The extra solution was removed by using blotting paper. Lastly, the thin film on the SEM grid was placed under mercury lamp for 5 min for complete drying purpose.

### 2.5. Antioxidant Activity

#### 2.5.1. DPPH Antioxidant Assay

Different concentrations (50, 100, 150, 200, 250, and 300 *μ*g/mL) of LAAgNPs and plant extracts were prepared from the stock solution through serial dilution (1 mg/mL). The mixture was incubated for 30 min at rt. The decrease in absorbance reading was measured at 517 nm using spectrophotometer. Ascorbic acid (3), which is known as an antioxidant was used as reference standard. The assay was performed in triplicate. The free radical scavenging activity was calculated as the percentage inhibition.

(1) $$\begin{array}{c} \text{Free radical scavenging activity}\%=\frac{\left[\left({\text{A}}_{\text{Control}}–{\text{A}}_{\text{Sample}}\right)\right]}{\left({\text{A}}_{\text{Control}}\right)\;}\times 100. \end{array}$$

#### 2.5.2. ABTS Antioxidant Assay

The final reaction mixture (1 mL) of standard and extracts comprised of 950 *μ*L of ABTS solution and 50 *μ*L of the sample. This reaction solution was vortexed for 10 sec. The UV-Vis readings were taken at 734 nm to measure absorbance, the result was compared with control ABTS solution, ascorbic acid (3) was used as reference standard, and the percentage inhibition was calculated (Equation (1)).

### 2.6. Antibacterial Activity

*In vitro* antibacterial activity of synthesised nanoparticles and leaf extracts was analysed by Kirby-Bauer technique. Here, three Gram-positive bacteria (*Bacillus subtilis*, *Staphylococcus aureus*, and *Streptococcus*) and two Gram-negative bacteria (*Escherichia coli* and *Shigella boydii*) were used for antibacterial assay. Initially, 20 *μ*L of both plant extracts and silver nanoparticles was pipetted into 6 mm of sterile antibacterial discs. The impregnated discs were left to dry at 30°C for 30 min. Prepared bacterial suspension solution was spread on NA agar by using sterile cotton swab, then dried the infused sterile discs with plant extracts; and silver nanoparticles were placed on NA agar. The plates were left to incubate at rt for 24 h, and ampicillin (4) was used as standard.

### 2.7. Anticancer Activity

#### 2.7.1. Cell Culture Condition

Dulbecco's modified Eagle's medium (DMEM) was used to propagate the cells which is supplemented with 10% fetal bovine serum and 1 unit/mL antibiotic penicillin/streptomycin. LAAgNP extract-mediated silver nanoparticles stock (10 mg/mL) was prepared in dimethyl sulfoxide (DMSO). Different types of concentrations were prepared which are of 10, 25, 50, 75, and 100 *μ*g/mL in culture medium for experimental purposes.

#### 2.7.2. Determination of Cell Viability by MTT Assay

Human glioma cells (DBTRG and U87) and human breast adenocarcinoma cells (MCF-7 and MDA-MB-231) with 0.5 − 1.0 × 10^4^ concentration were cultured with LAAgNPs for 24 h and were maintained at 37°C in 5%CO_2_ humidified atmosphere. The number of viable cells in both the samples was determined by MTT assay. The absorbance was recorded at 570 nm wavelength and the cell viability percentage against LAAgNPs concentrations were determined in the form of IC_50_, tamoxifen (5) was used as reference standard for activity against DBTRG and U87cell lines, and gefitinib (6) was used as reference standard for activity against MCF-7 and MDA-MB-231 cell lines.

### 2.8. Molecular Docking Studies

The potential bioactivity of the two FOG ligands (Cosmosioside and Cynaroside) has been mechanistically investigated from the molecular docking studies by predicting effective interactions against selected proteins. In execution, the protein crystal structures are obtained in PDB form from protein data bank repository and removed the unnecessary bound ligands, cofactors, and water molecules from their vicinity. The .mol2 and .pdb files of the corresponding FOG ligands were generated from ChemBioOffice 14.0 (Chem3D Pro) software and performed docking on SwissDock software [39]. The best outfit interactions optimized with energy minima at a gradient of 0.100 of root mean standard deviation were captured on UCSF Chimera by envisaging the best binding modes [40]. The binding energies of two FOG ligands interacted with the corresponding target protein receptors in chain A of 3NM8 (oxidoreductase, tyrosinase complex) for DPPH antioxidant activity, chain A of 1DNU (oxidoreductase, myeloperoxidase-thiocyanate complex) for ABTS antioxidant activity, chain A of 5FGK (transferase, cyclin-dependent kinase 8 associated with cyclin C) for gram positive bacteria (B. subtilis, S. aureus, and S. felicis in the current study), chain A of 1AB4 (topoisomerase, the N-terminal 59 kDa fragment of Gyrase A) from gram negative bacteria (E. coli and S. boydii in the current study), chain A of 4GBD (lyase, adenosine deaminase complex) for MCF-7 anticancer activity, chain C of 5FI2 (hydrolase, kidney glutaminase isoform C complex of UPGL 00009 inhibitor) for MDA-MB-231 anticancer activity, chain A of 1D5R (hydrolase, PTEN tumor suppressor) for U87 anticancer activity; and chain B of 5TIJ (lyase, human enolase 2 complex) for DBTRG anticancer activity have predicted in the macromolecular environment and also compared with their reference standards viz., ascorbic acid, ampicillin, tamoxifen, and gefetinib.

### 2.9. ADMET Properties

[41] The ADMET properties of 1 and 2 have been predicted from preADMET online server [42] to comprehend their biocapabilities like *in vitro* Caco-2 cell permeability, *in vivo* blood-brain barrier (BBB) penetration, *in vitro* Maden Darby Canine Kidney (MDCK) cell permeability, human intestinal absorption (%HIA), and *in vitro* plasma protein binding (PPB) properties. In extension mutagenic, tumarogenic, reproductive, and irritant effects have also been predicted to establish the detailed toxicity analysis for Cosmosioside and Cynaroside. The BBB deals with the intensely bound endothelial cells which oblige a compound's proficiency to be passed into the bloodstream through the administered route. The analysis of BBB penetration rate (BBB = [Brain]/[Blood]) helps to examine the capability of a compound to penetrate over blood-brain barrier, which is vital in allocating central nervous system (CNS) activity to the biological properties of a compound. The compounds with BBB penetration rate > 0.40 are passable through the BBB and are denoted as CNS active; on contrary, the compounds with BBB penetration rate < 0.40 are unable to pass through the BBB and are denoted as CNS inactive. Likewise, human colon adenocarcinoma-based Caco-2 cells that are associated with intestinal epithelium system in multiple drug transportation pathways like transcellular, paracellular, and active efflux transports are assessed by in vitro Caco-2 permeability value as the value is <4 is poor permeable, value in the range of 4-70 is moderately permeable, and value >70 is extremely permeable and is certainly transported to cellular cite in the biochemical processes. Furthermore, the degree of plasma protein binding (PPB) influences the level of distribution of compound unbound in body tissues and infers about unbound quantity of the compound that has been distributed in the active cellular sites and then stimulated further to metabolize and then excreted from the system. The in vitro PPB percentage > 90% classifies the compounds under study as strongly bound and *in vitro* PPB percentage < 90% classifies the compounds under study as weakly bound and eventually replicates its action as well as proficiency. In addition, the MDCK cell system is considered as a sensible tool to predict the prompt permeable compounds and determine their capability as greater the life span of Caco-2 cells than the cellular life span consequences for its high correspondence. Here, the compounds with *in vitro* MDCK permeability value < 25 are poor permeable, and compounds with in vitro MDCK permeability value in the range of 25-500 are good permeable. In addition, the percentage of HIA is considered as the percentage of an orally administered compound with significant bioavailability into the hepatic portal vein by absorption in relation to total content that excreted through bile, urine, and feces. Compounds with the percentage of HIA in the range of 0-20 are of identified with poor absorbance, 20-70 are of identified with moderate absorbance, and 70-100 are of identified with good absorbance. The consideration of toxicology properties of a compound with its structure greatly helps to design them with bioactivity. The negative toxicology value of a compound affords it as a safer drug works against mutagenicity, carcinogenicity, and human ether-a-go-go-related gene (HERG) channel inhibition.

### 2.10. QSAR Studies

Many drug candidates suffer to clear the clinical trials stage due to their inadequate absorption, distribution, metabolism, excretion, and toxic potentialities, where the worthy oral bioavailability made them as potential with right poise of partitioning and solubility. Computationally, Lipinski's rule of five [43] helps to screen newer molecules to affirm their potentiality based on the parameters like (i) molecular weight ≤ 500da, (ii) number of hydrogen bond donors ≤ 5, (iii) number of hydrogen bond acceptors ≤ 10, (iv) logP (octanol/water partition coefficient) ≤ 5, and (v) molar refractivity from 40 to 130. Likely the Lipinski parameters, Molinspiration [44] helps to predict Veber parameters (number of rotatable bonds and total polar surface area in addition to Lipinski parameters) and other parameters like Van der Waals volume, number of hydrophobic atoms, solubility, density, percentage of absorption, and octanol to water partition coefficient, which help to testify the structural sensitivity of the compound under study.

### 2.11. Bioactivity and Toxicity Risk Studies

The bioactivity and toxicity risk studies of compounds under study have been assessed on molinspiration online server [44] where physicochemical properties were explored on molinspiration v2018.10 engine, and biochemical properties were explored on molinspiration v2018.03 engine. This exploration revealed bioactivity properties like G protein-coupled receptor (GPCR) ligand property, kinase inhibition (KI) property, ion channel modulator (ICM) capability, nuclear receptor ligand (NRL) interactions, enzyme inhibitor (EI) properties, and protease inhibitor (PI) properties. Similarly explored the drug-likeness and drug scores along with the toxicity risks like tumorigenic, mutagenic, reproductive and irritant effects and proved that the compound 1 and 2 (Cosmosioside (1, Apigenin-7-glucoside) Cynaroside (2, Luteolin-7-glucoside) as safer drugs as predicted the results with Osiris online property explorer toolkit [45]. These predictions helped to understand physicochemical interactions of the compounds under study against their targets and ultimately helped to defining their drug properties.

### 2.12. Statistical Analysis

The results were expressed as the mean ± standard deviation of triplicates. Statistical analysis was performed using one-way analysis of variance (ANOVA) followed by Tukey's test. *P* < 0.05 was considered statistically significant.

## 3. Results and Discussion

### 3.1. Chemistry

#### 3.1.1. Observation of Colour Changes of Silver Nanoparticles

**Figure 2:**
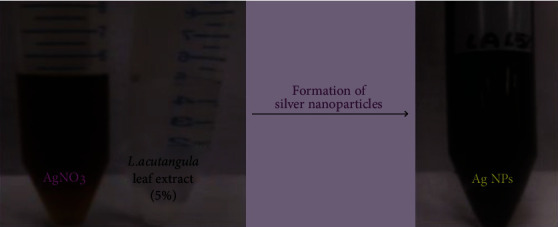
Formation of silver nanoparticles using 5% of *LA* leaf extract.

The colour of *LA* leaf extract was changed from light colour to brown colour (Figure 2), indicating the synthesis of silver nanoparticles. The noticeable colour change in *LA* leaf extract was mainly due to the reduction of Ag^+^ ions to Ag^0^ atoms (Equation (2)). Bounteous biomolecules present in the leaf extracts act as natural reducing agents and the reduction reaction can be summarised as follows.

(2) $$\begin{array}{c} \text{Plant Extract}+\text{AgNO}3⟶\text{Ag nanoparticles}. \end{array}$$

In this connection, previous studies showed similar colour changes to form dark brown colour [21, 46, 47]. It was confirmed that concentrations of plant extracts are one of the significant aspects that influence the rate of synthesis of silver nanoparticles. Higher intensity of colour was spotted as the concentrations increased from 1.0, 2.5, and 5.0%. This could be a result of higher content of the biomolecules that reacted as reducing agents in silver reduction process. Uniform results had also been noticed in the leaves of *Luffa acutangula* in synthesising silver nanoparticles [22].

#### 3.1.2. UV-Vis Spectral Studies of LA Silver Nanoparticles

**Figure 3:**
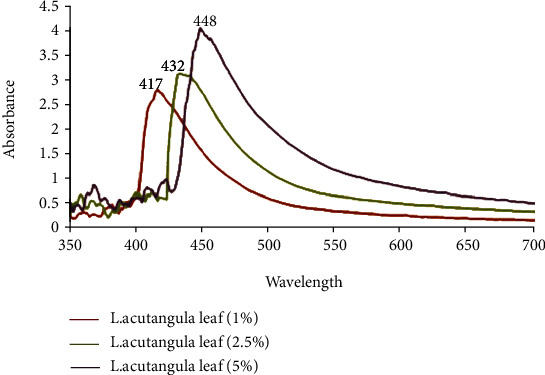
UV-Vis spectrum of LAAgNPs at various concentrations.

Time interval to measure the absorption peak was 30-150 sec. At 150 sec, the highest peak was observed for all the different concentrations, and all the high adsorption peaks were in the range of standard adsorption of silver nanoparticles. The spectral peaks were recorded at 417, 432, and 448 nm for different concentrations of biologically synthesised nanoparticles at 1.0, 2.5, and 5.0%, respectively (Figure 3).

There are no qualms that silver nanoparticles achieved the highest peak as cause of Surface Plasmon Resonance (SPR) adsorption band. Free electrons of biologically synthesised silver nanoparticles promote the generation of SPR band through coalescing the vibrations of electrons in resonance with the light wave [48]. The aspects like size and shape of the nanoparticles, type of biomolecules existing in the plant extracts, silver nitrate concentration, and amount of extracts have influenced the SPR banding patterns.

#### 3.1.3. XRD Analysis

**Figure 4:**
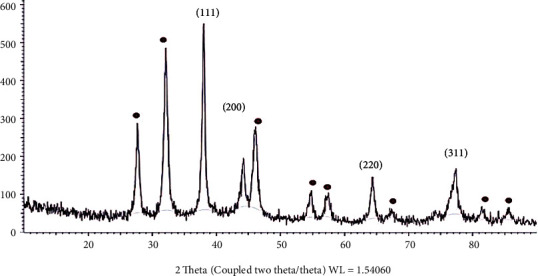
XRD graph of synthesised LAAgNPs (5%).

X-ray diffraction analysis is an advanced method to figure out the crystalline nature of metallic nanoparticles. As shown in Figure 4, the peaks at 2*θ* values of 38°, 44°, 64°, and 77° are reflecting (111), (200), (220), and (311) lattice plans for silver, respectively. The present result clearly illustrates that the biologically synthesised silver nanoparticles are in crystalline nature and face-centered cubic (fcc) shape. The studies on carob and olive leaf extract confirmed the shape of the synthesised nanoparticles as fcc [49, 50]. Debye Scherrer's equation (Equation (3)) was used to calculate the average particular size of the silver nanoparticles synthesised by present biological method, where *β* is the full width at half maximum of the diffraction peak, *t* is the mean crystalline size, *θ* is the centre angle of the peak, and *λ* is the wavelength of X-ray source. The recognized crystalline size of LAAgNPs is 44 nm. This clearly illustrates that LAAgNPs is nanocrystalline shape. Similar results have been reported on the biologically synthesised AgNPs using *Pulicaria glutinosa* plant extract showed average crystalline size of 42 nm [13].

(3) $$\begin{array}{c} t=\frac{0.89\lambda }{\beta cos\theta } \end{array}$$

#### 3.1.4. FTIR-ATR Analysis

**Figure 5:**
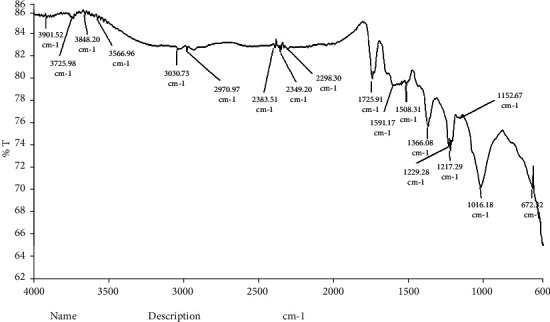
FTIR–ATR graph of synthesised LAAgNPs.

The interaction between biologically synthesised nanoparticles and biological molecules of aqueous leaf extracts of *LA* can be understood from the FTIR-ATR spectrum. In Figure 5, the absorption peaks at 3030.73 and 2970.97 cm^−1^ represent the O-H groups in alcohols, phenols, and C-H stretching of alkenes amide I or proteins [26]. Band appearing at 2383.51 and 2349.20 cm^−1^ denotes C-O groups. Further, the adsorption peaks at 2298.3 and 1508.31 cm^−1^ reflect the functional groups of –C=C- group and C=C of amide II groups, respectively ^12^. Specific IR bands at 1366.08, 1152.67, 1229.28, and 1217.29 cm^−1^ attribute to C-H alkenes and C-N stretching vibration of aliphatic amines, respectively [26]. Absorption peaks at 1016.18 and 672.32 cm^−1^ assign the presence of ether linkage and aromatic hydrocarbon [51, 52]. A similar result was reported for phenols, flavonoids, alkaloids, and proteins in plant extracts lead to stabilization and synthesis of AgNPs [50]. In the present study, the stretching vibrations of silver nanoparticles of *LA* indicated different proteins and terpenoids in aqueous extracts and enhanced the bioreduction of Ag ions. A previous study reported that *LA* leaves possess numerous molecules such as alkaloids, flavonoids, saponins, and glycosides [53]. These functional groups in aqueous leaf extracts of *LA* facilitate capping and reduction process of Ag ions. In the present study, the stretching vibrations of green mediated synthesised silver nanoparticles using *LA* indicated different proteins and terpenoids in aqueous extracts and enhanced the bioreduction of Ag ions. A previous study reported that *LA* leaves possess numerous molecules such as alkaloids, flavonoids, saponins, and glycosides [53]. These functional groups in aqueous leaf extracts of *LA* facilitate capping and reduction process of Ag ions.

#### 3.1.5. SEM Analysis

**Figure 6:**
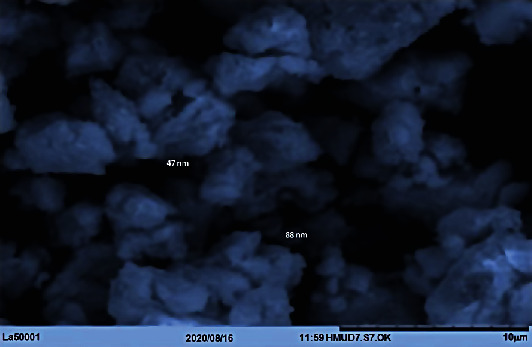
The SEM micrograph of synthesised LAAgNPs (5%).

The biosynthesised LAAgNPs were morphologically visualized on scanning electron microscopy (SEM) and identified as uniform and spherical in shape with 10 *μ*m size under 7000× magnification (Figure 6). However, the structure of all the AgNPs could be observed more clearly at higher magnification. Further, the overall SEM image is attributed due to electrostatic interaction between bioorganic capping molecules attached on the AgNPs surface. Several factors such as aggregation of the smaller ones and SEM measurements could influence the formation of larger AgNPs [11].

### 3.2. Antioxidant Activity

#### 3.2.1. DPPH Antioxidant Assay

**Figure 7:**
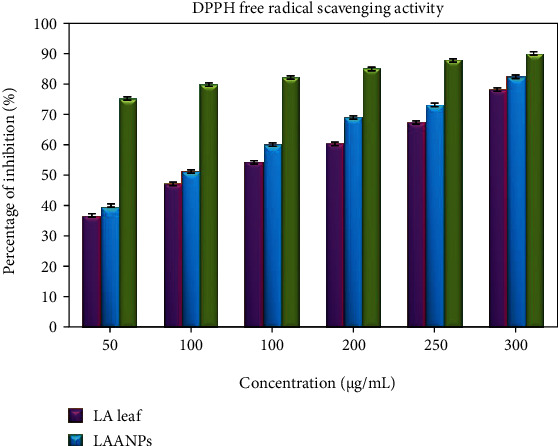
The DPPH free radical scavenging activity.

DPPH (1,1-diphenyl-2-picrylhydrazyl) free radical scavenging activity [54] was studied on leaf extracts and AgNPs from *LA* in this study. AA was chosen as positive control for comparison purposes. The *LA* leaf extract revealed free radical scavenging action by 37.1% to 79.1% at 50 *μ*g/mL to 300 *μ*g/mL concentrations, respectively. Further, biologically synthesised LAAgNPs demonstrated free radical scavenging activity from 39.9% to 83.2% at 50 *μ*g/mL to 300 *μ*g/mL concentrations (Figure 7). A similar result was reported for the biosynthesised AgNPs from *Syzygium cumini* (L.) seed extract exhibited high DPPH free radical scavenging activity [11] compared to *Argemone mexicana and Turnera ulmifolia* seed extracts [55].

The present data is in accordance with the result reported for the biosynthesised AgNPs from aqueous leaf extracts of *Terminalia mellueri*, *Terminalia catappa*, *Terminalia bellerica*, and *Terminalia bentazoe* showed high DPPH free radical scavenging activity (more than 80%) compared to leaf extracts in the range of 60%-70% [56]. The values represented are the mean ± S.D of triplicate sample significant level at (*P* < 0.05). The IC_50_ values of *LA* and LAAgNPs were 126.29 *μ*g/mL and 96.89 *μ*g/mL. The lower IC_50_ values indicate the greater tendency for antioxidant activity of the extracts. Similar activity was reported for the lower IC_50_ value of *Psidium guajava* extract and AgNPs from *Psidium guajava* was 110 *μ*g/mL and 80 *μ*g/mL, respectively [57].

#### 3.2.2. ABTS Antioxidant Assay

**Figure 8:**
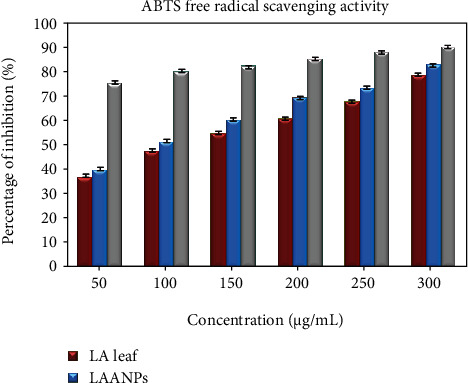
The ABTS free radical scavenging activity.

In the present study, ABTS free scavenging test was analysed on AgNPs and leaf extract of *LA*. ABTS^+·^ is considered as protonated radical which could readily accept electron from antioxidant compound and transfer its colour from blue to pink which was detected at 734 nm [58]. The leaf extract of *LA* showed the potential to scavenge the free radicals was found to be 43.8-82.9% at concentrations from 50-300 *μ*g/mL, respectively, whereas biologically synthesised AgNPs showed the activity as 47.9-85.2% at different concentrations from 50-300 *μ*g/mL, respectively (Figure 8). The values represented are the mean ± S.D of triplicate sample significant level at (*P* < 0.05). The IC_50_ value of standard AA is 31.42 *μ*g/mL which has proven that AA had higher scavenging activity with the lowest IC_50_ (*μ*g/mL). The IC_50_ values of *LA* and LAAgNPs were found as 100.96 and 76.0 *μ*g/mL, respectively. Similar activity was reported for the IC_50_ value of *Psidium guajava* extract (105 *μ*g/mL) and found higher than AgNPs from *Psidium guajava* (70 *μ*g/mL), respectively [57]. Similar ABTS radical scavenging action of biologically synthesised AgNPs was found in previous studies [59, 60].

### 3.3. Antibacterial Activity

There are no qualms that silver and silver-based compounds are the potential antibacterial or antimicrobial agents [61]. It has become compulsory to produce the safer substitutes for the currently available antimicrobial agents and also the antibiotics due to the high multidrug resistance problems [62]. The synthesis of metallic nanoparticles from the biological sources with potential antibacterial or antimicrobial properties has opened up a new avenue against multidrug resistance bacteria.

**Figure 9:**
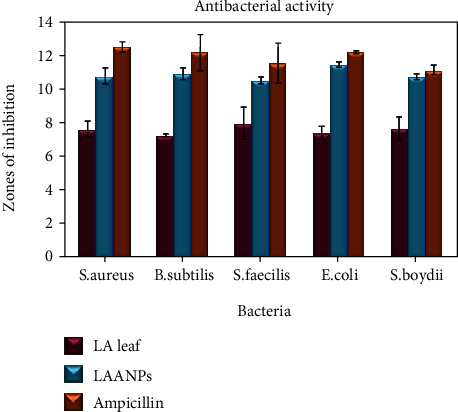
The results of antibacterial activity with zones of inhibition.

In the current study, human pathogenic microorganisms such as *B. subtilis*, *S. aureus, S. faecilis*, *E. coli*, and *S. boydii* were chosen to study the antibacterial efficacy of biologically synthesised silver nanoparticles. *LA* leaf aqueous extracts expressed potential antibacterial effect against both Gram-positive and Gram-negative bacterium. *LA* leaf extract expressed the highest inhibition which was seen in *S. faecilis* with 7.9 mm diameter, followed by *S. boydii* (7.6 mm), *S. aureus* (7.6 mm), *E. coli* (7.4 mm), and *B. subtilis* (7.2 mm). Moreover, biologically synthesised AgNPs revealed that the antibacterial efficacy of *LA* leaf extract was enhanced by inducing a higher zone of inhibition against the tested microorganisms. The silver nanoparticles from *LA* showed the highest inhibition against *E. coli*, Gram-negative bacteria. The zone of inhibition was recorded as 11.5 mm. Similarly, *LA* leaf-mediated silver nanoparticles did express high anticidal property by suppressing the growth of other microorganisms, *B. subtilis* (10.9 mm), *S. aurus* (10.8 mm), *S. boydii* (10.7 mm), and *S. faecilis* (10.5 mm) (Figure 9). Ampicillin was chosen as standard and positive control in this study. Ampicillin performed the highest inhibitory effect against all tested microorganisms compared to *LA* leaf extract and synthesised AgNPs.

**Figure 10:**
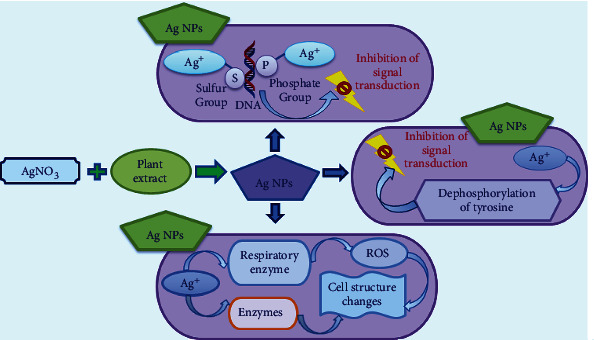
Different modes of antibacterial mechanisms induced by AgNPs in microbial cells.

The present study proved that gram-negative bacteria, *E. coli*, were more sensitive to the action of biologically synthesised silver nanoparticles compared to gram-positive bacteria. This is in accordance with the result stated by Kim and the coworkers [63]. Literature denoted the inhibitory effects of silver nanoparticles could be associated with characteristics of specific bacterial species. Naturally, gram-positive and gram-positive grouped bacteria have dissimilar membrane structure, especially the difference in thickness of peptidoglycan layer. The mild antibacterial features of synthesised silver nanoparticles in contradiction of gram-positive bacteria could be due to membrane structure [63]. The antibacterial mechanism of action of metallic nanoparticles is still not exactly explained and being unverified. However, several theories and possible mechanism(s) of actions of biologically and chemically synthesised silver nanoparticles have been reported with basic information [64]. The graphic representation (Figure 10) depicts the penetration of silver nanoparticles (AgNPs) into the cell and their different mode of antibacterial mechanisms. The reactivity begins with synthesis of silver nanoparticles using silver nitrate and selected plant extracts.

### 3.4. Anticancer Activity

**Figure 11:**
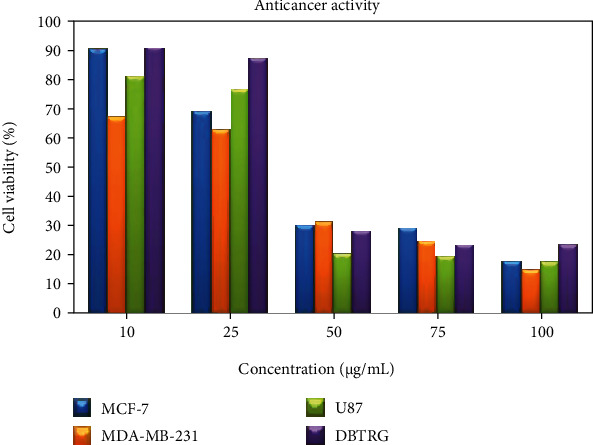
Cytotoxic activity of LAAgNPs against MCF-7, MDA-MB-231, U87, and DBTRG cell lines.

Plants contain several types of bioactive compounds that are ideally favorable for the drug development in anticancer therapy. Nowadays, researchers found that the plant-based medicines or drugs are safer and cost-effective when compared to the synthetic drugs [65]. *LA* is one of the herbal plants which belongs to a family of Cucurbitaceae and widely cultivated in Asia, India, Brazil, and USA [66]. Previously, itself isolated five major components of *LA*, a bioactive component among them named 1,8 dihydroxy-4-methylanthracene 9,10-dione (DHMA) was reported as potential antiproliferative agent against nonsmall cell lung cancer cells (NCI-H460). DHMA showed promising anticancer activities through inhibition of cell growth, generation of reactive oxygen species (ROS), and induction of p53-mediated apoptotic pathway against human nonsmall cell lung cancer cell line (NCI-H460) [67, 68]. Another study reported on the potential anticancer effect of *LA* on human colon cancer cell line HT29 cells [69]. *LA* seeds consist of ribosome inactivating proteins which were reported, and the study revealed the potential anticancer activity of luffaculin 1 and luffaculinin in human leukemia K562 cells [70]. Anticancer effects of AgNPs have been demonstrated in various cell models. It observed a dose-dependent cytotoxic effect of biosynthesized AgNPs from *Piper longum* extract in MCF-7 breast cancer cells [71]. Cytotoxic effects of AgNPs from other plant extracts such as *Iresine herbstii* and *Vitex negundo Linn* were demonstrated in HeLa (cervical) and HCT15 (colorectal) cancer cells, respectively [9, 25]. In the present study, LAAgNPs were tested against four human cancer cell lines, MCF-7, MDA-MB-231, DBTRG, and U87. The synthesised silver nanoparticles by *LA* leaf extract triggered a dose-dependent reduction in the cell proliferation with IC_50_ values ranging from 35-42 *μ*g/ml (Figure 11). There are several anticancer mechanisms that have been suggested based on previous studies. AgNPs tend to generate reactive oxidative species (ROS) intracellularly that results excess oxidative stress [72]. High oxidative stress inhibits chromosome inhibition and eventually damage cell cycle of tumor cells [73, 74]. Size independent property of AgNPs enhances cytotoxic effect against drug-resistant cancer cells [75]. In addition, cytotoxic effect can be as the result of poor angiogenesis and programmed cell death by AgNPs [76]. Further studies are needed to interpret the anticancer mechanism(s) of the biosynthesized AgNPs.

### 3.5. Molecular Docking Studies

The obtained *in vitro* antioxidant, antibacterial, and anticancer activity of FOGs have been additionally supported by investigating of ligand-protein binding interactions against the selected enzymatic proteins *viz*., 3NM8-Chain A (DPPH radical scavenging activity), 1DNU-Chain A (ABTS radical scavenging activity), 5FGK-Chain A (gram-positive bacterial activity), 1AB4-Chain A (gram-negative bacterial activity), 4GBD-Chain A (MCF-7 anticancer activity), 5FI2-Chain C (MDA-MB-231 anticancer activity), 1D5R-Chain A (U87 anticancer activity), and 5TIJ-Chain B (DBTRG anticancer activity); and docking postures and binding interactions were bestowed in Tables 1–12.

In view of antioxidant activity, the hydroxy groups (-OH) of FOGs bound with carbonyl groups (O=C) of aspartic acid and amino groups (-NH) of arginine in 3NM8 (Chain A) are responsible for DPPH radical scavenging activity; and binding of -OH of FOGs bound with O=C of aspartic acid and alanine, -NH of arginine, and -OH of tyrosine in 1DNU (Chain A) is responsible for ABTS radical scavenging activity. Concerning the antibacterial activity, the -OH of FOGs bound with C=O of aspartic acid, valine and glutamic acid, and -NH of aspartic acid and lysine in 5FGK (Chain A) is responsible for gram-positive bacterial activity; and binding of -OH in FOGs with C=O of aspartic acid, glutamine, histidine and phenyl alanine, and -NH of glutamine and lysine in 1AB4 (Chain A) is responsible for gram-negative bacterial activity. In relation to MCF-7 anticancer inhibition, the -OH of FOGs bound with C=O of aspartic acid and lysine, and -NH of arginine and isoleucine in 4GBD (Chain A) were identified as responsible. Coming to MDA-MB-231 anticancer inhibition, the -OH of FOGs bound with C=O of aspartic acid, cysteine and serine, -OH of tyrosine and glutamic acid, and -NH of asparagine in 5FI2 (Chain C) was identified as responsible.

Concerning the U87 anticancer inhibition, the -OH of FOGs bound with C=O of aspartic acid, and -NH of lysine, and tyrosine and arginine in 1D5R (chain A was identified as responsible). In aspects of DBTRG anticancer inhibition, the -OH of FOGs bound with C=O of aspartic acid and glutamic acid, -OH of glutamic acid and serine, and -NH of aspartic acid and serine in 5TIJ (chain B) was identified as responsible. The binding specificity studies have affirmed the promising ligand-protein binding interactions in between the hydroxy groups of the FOGs and aspartic acid of the concerned enzymatic proteins with a binding energy in the range of -9.2690 to -7.7955 KCal/mol.

### 3.6. ADMET Properties

The study of ADMET properties of the interested analytes under investigation helps to realize their physicochemical interactions [77]. The potentiality of a drug depends on its degree of absorption and in turn on its inherent bioavailability properties. Once a potential drug be absorbed and self-distributed in to muscles and organs by circulation through extracellular sites and hence lowers its plasma concentration individually, therefore, metabolizes *in vivo*, then, such metabolites will be distributed by the action of reduction and oxidation reactions by the enzymatic action and work potentially on cellular systems, and ultimately the inert metabolites will be automatically excreted from kidneys. Such analysis of ADMET properties (Table 13) inferred us that the two FOGs are with 0.0373 and 0.0336 of BBB penetration potentiality confirms their CNS significance and esteems their superior permeability and their *in vivo* distribution. Further, it is supported on the ground of the *in vitro* Caco-2 cell permeability held with 7.2167 and 4.8722 nm/sec, respectively, which enables their robust permeability to bind to plasma proteins and to penetrate in to the BBB system. The *in vitro* PPB efficiency with 73.43 and 73.27 respective percentages approves their robust binding capability to plasma proteins. The *in vitro* MDCK cell permeability with 0.6424 and 0.7567 nm/sec empowers their strong permeability. The %HIA with 47.1059 and 25.1651 supports their interactions with targeted domains of the cells. The negative magnitudes of the toxicity calculations that designate FOGs are nontoxic and with safer drug properties. In ultimate, ADMET analysis of the two FOGs has greatly manifested their potential physicochemical interactions and drug-likeness.

### 3.7. QSAR Studies

QSAR results (Table 14) indicate that FOGs under study with molecular weights 432.38 and 448.38 (less than 500 Daltons) have confirmed their greater permeability *via* cell membranes with log *P* values of 0.68 and 0.19 (less than 5). Correspondingly, the numbers of hydrogen bond acceptors and donors have also obeyed the limitations. The molecular refractivity values with 107.46 and 109.27 cm^3^/mol have aligned in the standard range (40-130 cm^3^/mol) and confirmed that the two FOGs are obeying the Lipinski rule of five and are designated as significant oral active drugs. On the other hand, the total polar surface area bankrolled by the addition of polar surface area of the atoms like oxygen, nitrogen, and hydrogen [78], for the two FOGs are with 170.05 and 190.28 A°^2^ obeying the limitations; and the number of total rotatable bonds in FOGs are 4 in number and obeying its potential boundaries; in complementary to obeying of the Lipinski rule, these two accounts for the validation of the Veber's rule pertaining to the two FOGs of the study. Henceforth, these FOGs are admired to be absorbed, diffused, and transported certainly and ascertained as oral administrable drugs. The TPSA is greatly correlated with the hydrogen bonding of a molecule and is complemented with transport properties of a drug through the membranes, and hence, also accounts for the BBB penetrability [79]. Furthermore, density with 1.642 and 1.713 gm/cc, solubility with -2.74 and -2.45, and *Van der Waals* volume with 356.17 and 364.19Å^3^, respectively, ascertain the safer and potential drug-likeness of the FOGs. In ultimate, this study greatly helped in accepting the physicochemical interactions of FOGs with the anticipated targets; and in defining their drug properties by complementing with bioactivity and toxicity risks studies, where the ligand interactions and enzyme inhibition properties along with the like drug-likeness and drug scores will be evaluated.

### 3.8. Bioactivity and Toxicity Risk Studies

The bioactivity and toxicity risk exploration studies of the FOGs have shown their bioactivity properties *viz*., GPCR ligand property, ion channel modulator, kinase inhibitor, nuclear receptor ligand interactions, protease inhibitor, and enzyme inhibitor interactions; and the drug properties like drug-likeness and drug score and established as potential nontoxic drugs (Table 15). This molinspiration exploration comprehensively assists us to explore the cheminformatics of the molecules under investigation by correlating with the *in vitro* and *in vivo* results database of the recognized drugs basing on the functional group similarities in mutual. The drug property exploration of the two FOGs has evidenced for their safer drug properties as they are with no risks of tumorigenicity, irritant effects, mutagenicity, and shown no effect on reproductive system. The positive magnitude of the drug-likeness value represents that the scrutinized molecule comprises the significant fragments that are present in the established commercial drugs [45]. Drug-likeness is an significant factor which helps in understanding the kinesis of a molecule from the site of administration to the bloodstream, hence, its good solubility accounts for good absorption and assures the drug-likeness [80]. Similarly, drug score is also a complementary parameter of the drug-likeness and helps to assure to decide molecule's drug potentiality. Hence, the present investigation reveals that all the properties of the bioactivity and toxicity risk studies are up to the potential limits of the safe drugs and ascertains the FOGs as the drug-like compounds.

## 4. Conclusions

*Luffa acutangula* is one of the regularly used plants with various secondary metabolites such as polyphenols and flavonoids, which possesses biological and pharmacological activities. Here in this study, the aim is to test the biogenically synthesised nanoparticles for their biological activities including antibacterial, antioxidant, and anticancer activities. The results revealed that the silver nanoparticles of *Luffa acutangula* leaf extract enriched with its inherent flavonoid *O*-glycosides (FOGs, *viz.,* Cosmosioside (1, Apigenin-7-glucoside) and from -9.2690 to -7.8306 for Cynaroside (2, Luteolin-7-glucoside)) prepared by green biosynthetic approach. The biogenically synthesised silver nanoparticles found to be significant against bacteria and cancer cell lines which clearly show antibacterial and anticancer activities. Antioxidants play an important in reducing the oxidative stress and diminishing the growth of the cancerous cell. The results showed that AgNPs showed potential antioxidant activity. The profound studies performed based on the molecular docking analysis have revealed that the FOGs are identified as antagonists of aspartic acid receptor of enzymatic proteins referenced based on the microorganisms, cell lines, and oxidizing agents considered for the *in vitro* studies. Furthermore, QSAR, ADMET properties showed them as prospective drugs. The results validated that AgNPs could be potential agents to treat various types of cancers and boosting the immune system functions. Nevertheless, future studies with *in vivo* toxicological studies with clear mechanism of action and the pharmacodynamics studies of LAAgNPs would shed the light more thoroughly to show the possible mechanisms for anticancer activities.

## Figures and Tables

**Table 1:** Potential ligand-protein molecular docking bindings of Cosmosioside (1) with identified proteins.

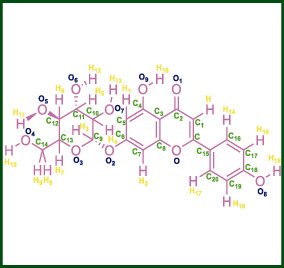	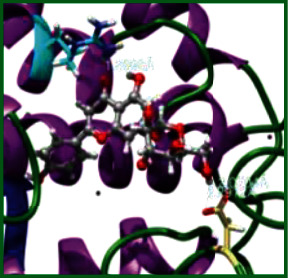	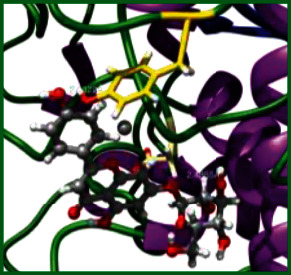
Cosmosioside (1, Apigenin-7-glucoside, C_21_H_20_O_10_)	3NM8 (chain A)-oxidoreductase-tyrosinase	1DNU (chain A)-oxidoreductase-myeloperoxidase-thiocyanate complex
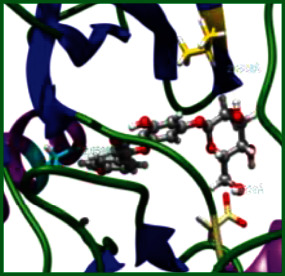	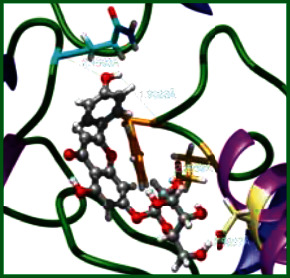	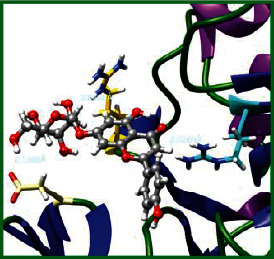
5FGK (chain A)-*A Transferase Enzymatic Protein*-CDK8-associated CycC	1AB4 (chain A)-*A Topoisomerase Enzymatic Protein*-59 KDA fragment of gyrase A	4GBD (chain A)–Lyase-adenosine deaminase
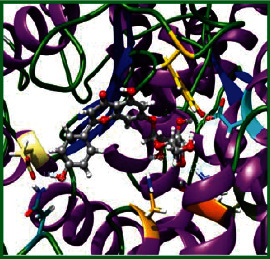	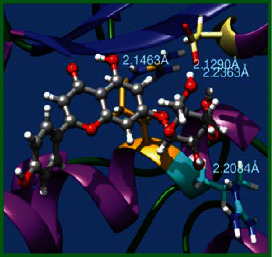	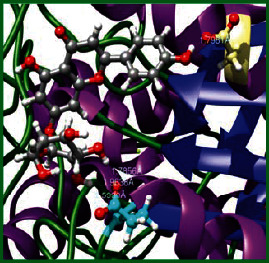
5FI2 (chain C)-*Hydrolase* Inhibitor Enzymatic Protein -GAC in complex UPGL 00009 inhibitor	1D5R (chain A)-*A Hydrolase Enzymatic Protein*-PTEN tumor suppressor	5TIJ (chain B)-A *Lyase* Enzymatic Protein-human enolase 2

**Table 2:** Potential ligand-protein molecular docking interactions of Cosmosioside (1) with identified proteins.

Enzymatic protein	Cluster number	Cluster element	BE (KCal/mol)	HB	HBL atoms	HBR atoms	Binding interactions	BL (A°)	BA (°)	H.B. type
3NM8 (chain A) - *oxidoreductase* - tyrosinase	0	7	-8.2618	3	2	3	Ligand[O_(4)_-H_(10)_]-------ASP55(OCOH)	2.0704	54.6126^##^	Acceptor
							Ligand[O_(4)_-H_(10)_]-----ASP55[O(H)CO]	2.2912		Acceptor
							Ligand[C_(2)_-O_(1)_]-----------ARG209(HN)	2.8998	—	Donor

1DNU (chain A) - *oxidoreductase* - myeloperoxidase-thiocyanate complex	30	7	-7.9463	2	2	2	Ligand[O_(7)_-H_(13)_]-----------ASP172(OC)	2.4855	—	Acceptor
							Ligand[O_(8)_-H_(18)_]----------TYR316(OH)	2.2322	—	Acceptor

5FGK (chain A) - *transferase* - CDK8 associated CycC	0	0	-9.1645	3	3	3	Ligand[O_(4)_-H_(10)_]------ASP103(OCOH)	1.9270	—	Acceptor
							Ligand[O_(6)_-H_(12)_]------------VAL27(OC)	2.4636	—	Acceptor
							Ligand[O_(8)_-H_(18)_]-------GLU66(OCOH)	2.0588	—	Acceptor

1AB4 (chain A) - *topoisomerase* -59KDA fragment of gyrase A	0	0	-8.2294	4	4	4	Ligand[O_(4)_-H_(10)_]-------ASP87(OCOH)	2.5627	—	Acceptor
							Ligand[O_(7)_-H_(13)_]-----GLN94(OCNH_2_)	2.2952	—	Acceptor
							Ligand[O_(8)_-H_(18)_]------------PHE96(OC)	1.9022	—	Acceptor
							Ligand[H_(18)_-O_(8)_]----------GLN267(HN)	2.1500	—	Donor

4GBD (chain A) - *Lyase* - adenosine deaminase	8	1	-7.7955	3	3	3	Ligand[O_(4)_-H_(10)_]-------ASP36(OCOH)	2.1348	—	Acceptor
							Ligand[H_(13)_-O_(7)_]---------ARG411(HN)	2.0486	—	Donor
							Ligand[C_(2)_-O_(1)_]-----------ARG149(HN)	2.5263	—	Donor

5FI2 (chain C) - *hydrolase inhibitor* - GAC in complex UPGL 00009 inhibitor	0	0	-8.8921	7	6	7	Ligand[O_(8)_-H_(18)_]------ASP446(OCOH)	1.8467	—	Acceptor
							Ligand[O_(4)_-H_(10)_]----------TYR248(OH)	2.1513	—	Acceptor
							Ligand[O_(6)_-H_(12)_]----------GLU380(OH)	1.7125	—	Acceptor
							Ligand[H_(18)_-O_(8)_]------ASN318(HNOC)	2.2940	—	Donor
							Ligand[C_(9)_-O_(3)_-C_(13)_]-ASN334(HNOC)	2.6215	—	Donor
							Ligand[H_(11)_-O_(5)_]----------ASN387(HN)	2.1575	106.6382^@^	Donor
							Ligand[H_(11)_-O_(5)_]----------TYR413(HO)	2.4479		Donor

1D5R (chain A) - *hydrolase* - PTEN tumor suppressor	0	0	-8.7975	4	4	3	Ligand[O_(4)_-H_(10)_]------ASP324(OCOH)	2.2363	102.4531^$^	Acceptor
							Ligand[O_(6)_-H_(12)_]------ASP324(OCOH)	2.1290		Acceptor
							Ligand[H_(13)_-O_(7)_]----------ARG172(HN)	2.2084	—	Donor
							Ligand[H_(19)_-O_(9)_]----------ARG173(HN)	2.1463	—	Donor

5TIJ (chain B) - *lyase* - human enolase 2	5	7	-7.9044	4	3	3	Ligand[O_(8)_-H_(18)_]------ASP282(OCOH)	1.7981	—	Acceptor
							Ligand[O_(7)_-H_(13)_]---GLU219(O(H)OC)	2.5990	56.2469^#^	Acceptor
							Ligand[O_(7)_-H_(13)_]-----GLU219(OCOH)	1.9638		Acceptor
									69.7145^∗^	
							Ligand[O_(6)_-H_(12)_]-----GLU219(OCOH)	1.7856		Acceptor

^$^Ligand H_(10)_-ASP324(OCOH)-Ligand H_(12)_; ^∗^Ligand H_(13)_-GLU219(HOCO)- Ligand H_(12)_; ^##^ASP55[OC(H)O]-Ligand H_(10)_-ASP55(OCOH); HBL atoms: number of hydrogen bond ligand atoms; ^#^GLU219(HOCO)-Ligand H_(13)_-GLU219(O(H)OC); ^@^ASN387(NH)-Ligand O_(5)_-TYR413(HO); HB: number of hydrogen bonds; HBR Atoms: number of hydrogen bond receptor atoms; BE: binding energy; BL: bond length; BA: bond angle.

**Table 3:** Potential ligand-protein molecular docking bindings of Cynaroside (2) with identified proteins.

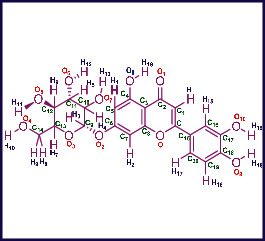	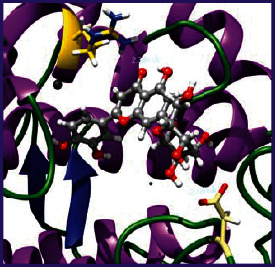	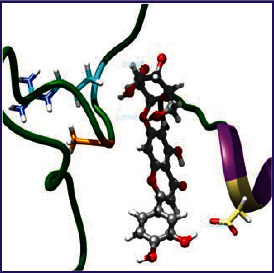
Cynaroside (2, luteolin-7-glucoside), C21H20O11)	3NM8 (chain A) - oxidoreductase - tyrosinase	1DNU (chain A) - oxidoreductase - myeloperoxiase-thiocyanate complex
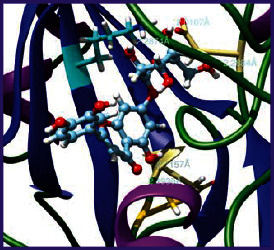	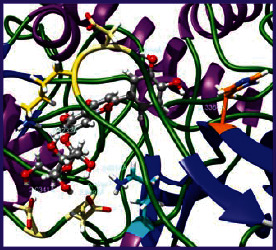	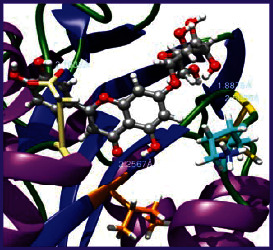
5FGK (chain A) - A Transferase Enzymatic Protein - CDK8 associated CycC	1AB4 (chain A) - A Topoisomerase Enzymatic Protein -59KDA fragment of gyrase A	4GBD (chain A) – lyase - adenosine deaminase
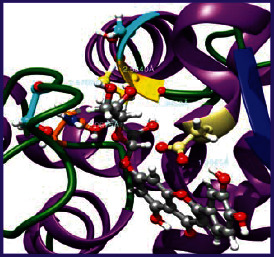	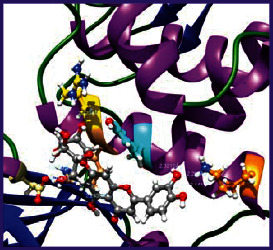	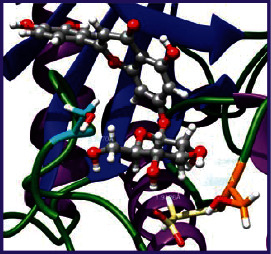
5FI2 (chain C) - Hydrolase Inhibitor Enzymatic Protein - GAC in complex UPGL 00009 inhibitor	1D5R (chain A) - A Hydrolase Enzymatic Protein - PTEN tumor suppressor	5TIJ (chain B) - A Lyase Enzymatic Protein -human enolase 2

**Table 4:** Potential ligand-protein molecular docking interactions of Cynaroside (2) with identified proteins.

Enzymatic protein	Cluster number	Cluster element	BE (KCal/mol)	HB	HBL atoms	HBR atoms	Binding interactions	BL(A°)	BA (°)	HB type
3NM8 (chain A) - *oxidoreductase* - tyrosinase	19	3	-7.9386	2	2	2	Ligand[O_(7)_-H_(13)_]----ASP324(OCOH)	2.1516	—	Acceptor
							Ligand[C_(2)_-O_(1)_]--------ARG209(HN)	2.3601	—	Donor

1DNU (chain A) - *oxidoreductase* - myeloperoxidase-thiocyanate complex	0	3	-8.3238	3	3	3	Ligand[O_(10)_-H_(19)_]---ASP324(OCOH)	1.9449	—	Acceptor
							Ligand[O_(7)_-H_(13)_]---------ARG27(HN)	2.0962	—	Acceptor
							Ligand[O_(6)_-H_(12)_]----------ALA24(OC)	2.2144	—	Acceptor

5FGK (chain A) - *transferase* - CDK8 associated CycC	18	12	-8.8514	5	5	5	Ligand[O_(5)_-H_(11)_]----ASP173(OCOH)	2.0107	—	Acceptor
							Ligand[C_(14)_-O_(4)_]--------ASP173(HN)	2.2884	—	Donor
							Ligand[C_(10)_-O_(7)_]----------LYS52(HN)	2.2874	—	Donor
							Ligand[H_(14)_-O_(8)_]---------LYS52(HN)	2.4930		Donor
							Ligand[O_(8)_-H_(14)_]---------ASP98(OC)	2.7157		Acceptor

1AB4 (chain A) - *topoisomerase* -59KDA fragment of gyrase A	1	3	-8.2753	7	7	6	Ligand[O_(5)_-H_(11)_]----ASP113(OCOH)	2.0095	49.2269^∗^	Acceptor
							Ligand[O_(7)_-H_(13)_]- --ASP113(OCOH)	2.6325		Acceptor
							Ligand[O_(4)_-H_(10)_]----ASP115(OCOH)	2.0341	—	Acceptor
							Ligand[O_(10)_-H_(19)_]-------ASP299(OC)	2.7050	—	Acceptor
							Ligand[C_(10)_-O_(7)_]--------LYS270(HN)	2.0314	—	Donor
							Ligand[C_(6)_-O_(2)_]---------LYS298(HN)	2.4233	—	Donor
							Ligand[O_(9)_-H_(18)_]-------HSD262(OC)	2.3361	—	Acceptor

4GBD (chain A) – *lyase* - adenosine deaminase	4	4	-7.8306	4	4	4	Ligand[O_(10)_-H_(19)_]--ASP215(OCOH)	2.1032	—	Acceptor
							Ligand[O_(6)_-H_(12)_]------LYS182(OC)	2.5865	—	Acceptor
							Ligand[O_(4)_-H_(10)_]--------LYS184(OC)	1.8875	—	Acceptor
							Ligand[H_(19)_-O_(9)_]---------ILE189(HN)	2.2567	—	Donor

5FI2 (Chain C) - *hydrolase inhibitor* - GAC in complex UPGL 00009 inhibitor	0	0	-9.1290	5	5	5	Ligand[O_(10)_-H_(19)_]---ASP446(OCOH)	1.9065	—	Acceptor
							Ligand[C_(11)_-O_(6)_]-------ASN330(HN)	2.0675	—	Donor
							Ligand[O_(5)_-H_(11)_]--------CYS462(O'C)	2.5840	—	Acceptor
							Ligand[O_(7)_-H_(13)_]---------CYS462(OC)	1.9953	—	Acceptor
							Ligand[O_(4)_-H_(10)_]---------SER313(OC)	2.5769	—	Acceptor

1D5R (chain A) - *hydrolase* - PTEN tumor suppressor	0	0	-9.2690	7	7	5	Ligand[O_(4)_-H_(10)_]----ASP324(OCOH)	1.9343	90.6238^$^	Acceptor
							Ligand[O_(6)_-H_(12)_]----ASP324(OCOH)	1.8831		Acceptor
							Ligand[C_(18)_-O_(9)_]---------LYS183(HN)	2.4417	74.4086^#^	Donor
							Ligand[C_(17)_-O_(10)_]--------LYS183(HN)	2.2624		Donor
							Ligand[O_(10)_-H_(19)_]--------TYR176(HN)	2.3215	—	Acceptor
							Ligand[C_(4)_-O_(8)_]---------ARG173(HN)	2.2435	—	Donor
							Ligand[O_(7)_-H_(13)_]--------ARG172(HN)	2.2234	—	Donor

5TIJ (chain B) -*lyase* - human enolase 2	24	1	-8.7736	5	4	5	Ligand[O_(5)_-H_(11)_]----ASP142(OCOH)	1.9126	—	Acceptor
							Ligand[O_(7)_-H_(13)_]---------ASP142(OC)	2.4464	—	Acceptor
							Ligand[C_(10)_-O_(7)_]---------ASP142(HN)	2.1296	48.5632^@^	Donor
							Ligand[C_(10)_-O_(7)_]---------SER141(HN)	2.3549		Donor
							Ligand[C_(14)_-O_(4)_]---------SER432(HO)	2.2870	—	Donor

**Table 5:** Potential ligand-protein molecular docking bindings of ascorbic acid (3) with identified proteins.

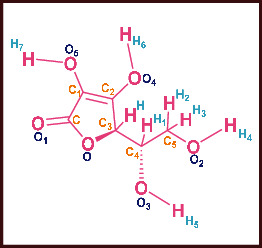	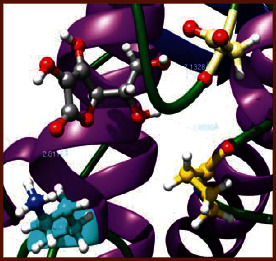	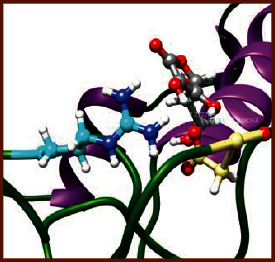
Ascorbic acid (3, hex-1-enofuranos-3-ulose, C_6_H_8_O_6_)	3NM8 (chain A) - oxidoreductase - tyrosinase	1DNU (chain A) - oxidoreductase - myeloperoxidase-thiocyanate complex

**Table 6:** Potential ligand-protein molecular docking interactions of ascorbic acid (3) with identified proteins.

Enzymatic protein	Cluster number	Cluster element	BE (KCal/mol)	No. of HB	No. of HBL atoms	No. of HBR atoms	Binding interactions	BL (A°)	BA (°)	HB type
3NM8 (chain A) - *oxidoreductase* - tyrosinase	1	4	-6.4837	3	3	3	Ligand[O_(2)_-H_(4)_]---ASP140(C=O)	2.1328	—	Acceptor
							Ligand[C-O_(1)_]--------LYS47(NH)	2.8116	—	Donor
							Ligand[O_(5)_-H_(3)_]---PRO219(C=O)	1.9298	—	Acceptor

1DNU (chain A) - *oxidoreductase* - myeloperoxidase-thiocyanate complex	31	2	-6.6112	4	3	3	Ligand[O_(4)_-H_(6)_]------ASP5(O=C)	2.3826	78.7795^∗^	Acceptor
							Ligand[O_(4)_-H_(6)_]----ASP5(OCOH)	1.9709		Acceptor
									61.7009^#^	
							Ligand[O_(5)_-H_(7)_]---ASP5(OCOH)	1.7722		Acceptor
							Ligand[H_(7)_-O_(5)_]-----ARG17(NH)	2.7187	—	Donor

^∗^ASP5(CO)-Ligand H_(6)_-ASP5(OCOH); ^#^Ligand H_(6)_-ASP5(OCOH)-Ligand H_(7)_.

**Table 7:** Potential ligand-protein molecular docking bindings of ampicillin (4) with identified proteins.

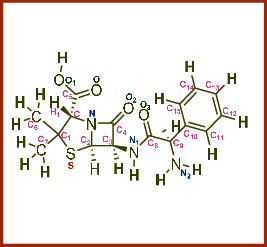	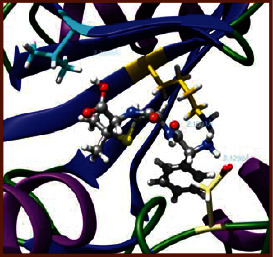	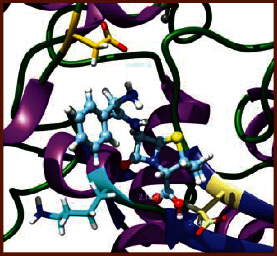
Ampicillin (4, aminobenzylpenicillin, C_16_H_19_N_3_O_4_S)	5FGK (chain A) - *A Transferase Enzymatic Protein* - CDK8 associated CycC	1AB4 (chain A) - *A Topoisomerase Enzymatic Protein* -59KDA fragment of gyrase A

**Table 8:** Potential ligand-protein molecular docking interactions of ampicillin (4) with identified proteins.

Enzymatic protein	Cluster number	Cluster element	BE (KCal/mol)	No. of HB	No. of HBL atoms	No. of HBR atoms	Binding interactions	BL (A°)	BA (°)	HB type
5FGK (chain A) - *transferase* - CDK8 associated CycC	5	0	-8.4767	3	3	3	Ligand[N_(2)_-H_(17)_]--ASP173(OCOH)	2.1290	—	Acceptor
							Ligand[C_(8)_-O_(3)_]----LYS52(HNCH_2_)	2.1803	—	Donor
							Ligand[O_(1)_-H_(3)_]--------VAL27(OC)	2.3334	—	Acceptor

1AB4 (chain A) - *topoisomerase* -59KDA fragment of gyrase A	1	4	-7.8486	3	3	3	Ligand[O_(1)_-H_(3)_]---ASP104(OCOH)	2.1401	—	Acceptor
							Ligand[N_(2)_-H_(18)_]--ASP515(OCOH)	2.4497	—	Acceptor
							Ligand[C_(5)_-O]-----------LYS129(HN)	2.1821	—	Donor

**Table 9:** Potential ligand-protein molecular docking bindings of tamoxifen (5) with identified proteins.

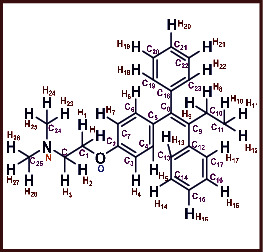	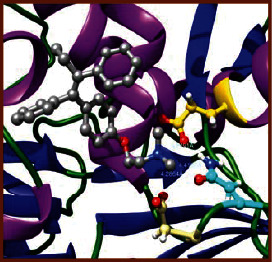	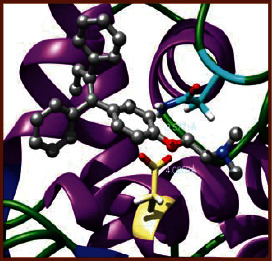
Tamoxifen (5, 1-*p*-*β*-dimethylaminoethoxyphenyl-trans-1,2-diphenylbut-1-ene, C_26_H_29_NO)	4GBD (chain A) – lyase - adenosine deaminase	5FI2 (chain C) - *Hydrolase* Inhibitor Enzymatic Protein -GAC in complex UPGL 00009 inhibitor

**Table 10:** Potential ligand-protein molecular docking interactions of tamoxifen (5) with identified proteins.

Enzymatic protein	Cluster number	Cluster element	BE (KCal/mol)	HB	HBL atoms	HBR atoms	Binding interactions	BL (A°)	BA (°)	HB type
4GBD (chain A) - *lyase* - adenosine deaminase	0	3	-7.7235	3	1	3	Ligand[C-N]-------ASP316(OCOH)	4.2864	39.9133^∗^	Acceptor
							Ligand[C-N]----------ASN314(O=C)	4.4192		Acceptor
									40.7597^$^	
							Ligand[C-N]----------GLU282(OCOH)	3.2491		Acceptor

5FI2 (Chain C) - *hydrolase inhibitor* - GAC in complex UPGL 00009 inhibitor	4	0	-7.5313	2	2	2	Ligand[C_(2)Ph_-O]----------ASP466(HN)	4.0253	104. 5635^#^	Donor
							Ligand[C_(2)Ph_-O]---------ASN318(HN)	3.5771		Donor

^∗^ASP316(HOCO)-Ligand[CN]-ASN314(OC); ^$^ASN314(CO)-Ligand[CN]-GLU282(OCOH); ^#^ASP466(NH)-Ligand[O]-ASN318(HN).

**Table 11:** Potential ligand-protein molecular docking bindings of gefitinib (6) with identified proteins.

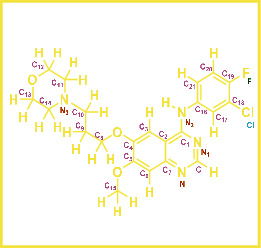	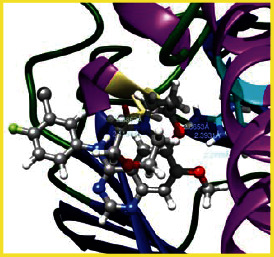	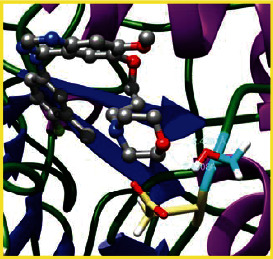
Gefitinib (6, N-(3-chloro-4-fluorophenyl)-7-methoxy-6-(3-morpholinopropoxy) quinazolin-4-amine, C_22_H_24_ClFN_4_O_3_)	1D5R (chain A) - *A Hydrolase Enzymatic Protein* - PTEN tumor suppressor	5TIJ (chain B) - A *Lyase* Enzymatic Protein - human enolase 2

**Table 12:** Potential ligand-protein molecular docking interactions of gefitinib (6) with identified proteins.

Enzymatic protein	Cluster number	Cluster element	BE (KCal/mol)	HB	HBL atoms	HBR atoms	Binding interactions	BL (A°)	BA (°)	HB type
1D5R (chain A) - *hydrolase* - PTEN tumor suppressor	0	0	-8.0338	5	3	4	Ligand[N_(2)_-H_(3)_]------ASP324(OCOH)	2.3863	54.6314^$^	Acceptor
							Ligand[N_(2)_-H_(3)_]------ASP324(OCOH)	2.1188		Acceptor
							Ligand[C_(5)_-O_(2)_]-------ARG173(HN=C)	2.2728	71.0798^#^	Donor
							Ligand[C_(4)_-O]---------ARG173(HN=C)	2.3931		Donor
									50.4066^∗^	
							Ligand[C_(4)_-O]--------ARG173(HN-CH)	2.8653		Donor

5TIJ (chain B) -*lyase* - human enolase 2	13	3	-7.8985	2	1	2	Ligand[C_(12)_-O_(1)_]----------ASP142(HN)	2.4308	52.5638^$^	Donor
							Ligand[C_(12)_-O_(1)_]----------SER141(HN)	2.4781		Donor

^$^ASP324(HOCO)-Ligand [H_(3)_]-ASP324(O(H)OC); ^∗^ARG173(C = HN)-Ligand[O]-ARG173(HN=CH); ^#^Ligand[O_(2)_]-ARG173(HN=C)-Ligand[O]; ASP142(NH)-Ligand[O_(1)_]-SER141(HN).

**Table 13:** ADMET properties the compounds 1-6.

Compounds	*In vivo* blood-brain barrier penetration (C. brain/C. blood)^a^	*In vitro* Caco-2 cell permeability (nm/sec)^b^	*In vitro* plasma protein binding (%)^c^	*In vitro* MDCK cell permeability (nm/sec)^d^	Human intestinal absorption (HIA, %)^e^	Toxicity^f^
Apigenin-7-glucoside (1)	0.0373	7.2167	73.4332	0.6424	47.1059	Negative
Luteolin-7-glucoside (2)	0.0336	4.8722	73.2796	0.7567	25.1651	Negative
Ampicillin (3)	0.0588	0.6307	36.1547	0.9376	81.4785	Negative
Ascorbic acid (4)	0.1173	2.4836	5.3035	0.8819	33.1572	Negative
Gefitinib (5)	0.0476	54.1474	80.7309	0.07737	96.6375	Negative
Tamoxifen (6)	14.1639	49.5448	94.7448	69.8462	100	Negative

^a^Blood − brain barrier (BBB) penetration = [brain]/[blood]; ^b^Caco-2 cells are derived from human colon adenocarcinoma and possess multiple drug transport pathways through intestinal epithelium; ^c^% of drug binds to plasma protein; ^d^MDCK cell system used as tool for rapid permeability screening; ^e^human intestinal absorption is the sum of bioavailability and absorption evaluated from ratio of excretion or cumulative excretion in urine, bile, and feces; ^f^*in vitro* Ames test by metabolic and nonmetabolic activated TA100 and TA1535 strains collected from rat liver homogenate.

**Table 14:** QSAR properties of the compounds 1-6.

Entry	Lipinski parameters						Veber parameters			Other parameters			
	MW	HB Don	HB Acc	logP (o/w)	MR	Lip. Vio.	TPSA	No. of RB	Veb. Vio.	No. of H	V. Volume	*ρ*	Solubility
Apigenin-7-glucoside (1)	432.38	10	6	0.68	107.46	0	170.05	4	0	20	356.17	1.642	-2.74
Luteolin-7-glucoside (2)	448.38	11	7	0.19	109.27	0	190.28	4	0	20	364.19	1.713	-2.45
Ampicillin (3)	349.41	7	4	-0.87	89.37	0	112.73	4	0	19	298.87	1.453	-1.57
Ascorbic acid (4)	176.12	6	4	-1.40	36.61	0	107.22	2	0	8	139.71	1.954	-0.35
Gefitinib (5)	446.91	7	1	4.19	118.38	0	68.75	8	0	24	385.07	1.322	-5.06
Tamoxifen (6)	371.52	2	0	6.06	119.87	0	12.47	8	0	29	376.13	1.042	-4.40

MW: molecular weight; HB Don: hydrogen bond donors; HB Acc: hydrogen bond acceptors; logP: octanol to water partition coefficient; MR: molecular refractivity (cm^3^/mol); Lip Vio: Lipinski violations; TPSA: total polar surface area; No. of RB: number of rotatable bonds; Veb Vio: Veber violations; No. of “H”: number of hydrophobic atoms; V. Volume: *Van der Waals* volume; *ρ*: density (gm/cc).

**Table 15:** Bioactivity scores, drug properties, and toxicity risks of the compounds 1-6.

Compounds	Structure	Bioactivity						Drug properties	
		GPCRL	ICM	KI	NRL	PI	EI	Drug-likeness	Toxicity risks
Apigenin-7-glucoside (1)	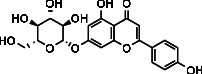	0.10	-0.01	0.14	0.31	0.02	0.43	2.29	0.44
Luteolin-7-glucoside (2)	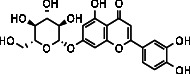	0.09	-0.02	0.15	0.27	-0.01	0.42	1.79	0.45
Ampicillin (3)	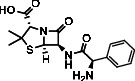	0.04	-0.47	-0.71	-0.61	0.87	0.25	10.72	0.91
Ascorbic acid (4)	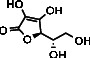	-0.53	-0.24	-1.09	-1.01	-0.81	0.20	0.02	0.74
Gefitinib (5)	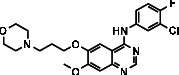	0.12	-0.04	0.66	-0.21	0.30	0.03	-2.62	0.28
Tamoxifen (6)	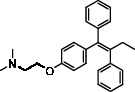	0.30	0.002	-0.01	0.57	0.04	0.32	6.3	0.35

GPCRL: G protein-coupled receptor ligand; ICM: ion channel modulator; KI: kinase inhibitor; NRL: nuclear receptor ligand; PI: protease inhibitor; EI: enzyme inhibitor.

## Data Availability

The data has been included in the manuscript.
